# The Influence of Y_2_O_3_ Dosage on the Performance of Fe60/WC Laser Cladding Coating

**DOI:** 10.3390/molecules30234598

**Published:** 2025-11-29

**Authors:** Haiyan Jiang, Dazhi Jiang, Chenguang Guo, Xiaodong Hong

**Affiliations:** 1 Basic Department, Liaoning Institute of Science and Technology, Benxi 117004, China; 2 School of Mechanical Engineering, Liaoning Technical University, Fuxin 123000, China; 3 School of Materials and Energy, Foshan University, Foshan 528000, China

**Keywords:** laser cladding coating, WC, Y_2_O_3_, wear resistance, corrosion resistance

## Abstract

To prepare a high-performance Fe-based laser cladding coating, herein, various Fe60/WC/Y_2_O_3_ coatings are deposited on the surface of 42CrMo steel plate via a laser cladding technique. The WC dosage is fixed as 10 wt%, while the dosage of Y_2_O_3_ ranges from 0 to 7.5 wt%. The influences of Y_2_O_3_ dosage on the coating hardness, wear resistance, and corrosion resistance are investigated. With the addition of Y_2_O_3_, the feature peak of WC disappears, and the peaks of M_23_C_6_ gradually weaken, indicating that Y_2_O_3_ promotes the decomposition of WC and suppresses the formation of new metal carbides. When the dosage of Y_2_O_3_ is 2.5 wt%, a grid-like structure is formed on the coating surface, suggesting uniform distribution of decomposed W within the Fe matrix. When the Y_2_O_3_ dosage exceeds 5 wt%, a large amount of CO_2_ gas is released, leading to an increase in surface pores. Through a comparison, the optimal dosage of Y_2_O_3_ is 2.5 wt%, and the resulting 3# coating has the highest hardness of 861.97 HV. Moreover, the 3# coating also shows the minimum friction coefficient and the minimum wear volume, reflecting its superior wear resistance. The polished coating serves as a working electrode, and the corrosion resistance is tested in 3.5% NaCl solution. The sample containing 2.5 wt% Y_2_O_3_ has the highest corrosion potential and the lowest corrosion current density, indicating excellent corrosion resistance. The enhanced performance is ascribed to the improved surface quality and the formation of a W-reinforced grid structure. The high-performance coating has promising application potential in material and component repair.

## 1. Introduction

Surface modification technology plays a crucial role in improving materials’ properties and extending their service lives [1]. Among the existing surface modification techniques, laser cladding coating technology is a process method in which a high-energy laser beam is engaged to rapidly melt cladding materials, such as alloy powder, ceramics, etc., onto the substrate surface [2]. This forms a metallurgical bonded surface coating, thereby significantly improving the wear resistance, corrosion resistance, and other properties of the substrate materials [3]. In terms of specific applications, laser cladding technology is a new technology with many economic benefits. It can prepare high-performance alloy surfaces on inexpensive metal substrates without affecting the properties of the substrate, reduce costs, and save precious and rare metal materials [4]. Therefore, researchers have attached great importance to research on and applications of laser cladding technology. In addition to studies on metal or alloy cladding coatings, various metal carbides have been widely used as reinforcing phases for fabricating carbide-reinforced metal-based cladding coatings, further enhancing the overall properties [5,6]. For example, WC is often used to enhance the performance of alloy coatings [7] owing to its high hardness and excellent wear resistance. Introducing WC into Fe-based coatings promotes the formation of a dense and uniform microstructure, effectively improving both the wear and corrosion resistance of the coating. However, an excessive amount of WC will compromise the surface quality of the sample and generate a large number of pores, thereby decreasing the coating performance [8]. To reduce the WC dosage and avoid performance degradation, appropriate amounts of rare earth oxides have been proven effective in significantly improving the wear and corrosion resistance of coatings [9,10]. In recent years, rare earth oxide Y_2_O_3_ has exhibited great potential for material modification owing to its high melting point and superior corrosion resistance [11]. When applied in Fe-based laser cladding coating, Y_2_O_3_ acts as a refiner, leading to enhanced hardness and wear resistance by grain refinement and microstructure optimization. For example, Li et al. [12] introduced TiB/TiC/Y_2_O_3_ reinforcement phases into a Ti-6Al-4V matrix using laser cladding technology and discussed the impact of Y_2_O_3_ on the coating microstructure and mechanical properties. The results demonstrated that the addition of Y_2_O_3_ refined the primary phase structure, resulting in the uniform distribution of the internal constituents. Furthermore, the coating containing Y_2_O_3_ exhibited improved resistance to micro-cutting and cracking. Liang et al. [13] fabricated Ni/WC/Y_2_O_3_ coatings on 316L stainless steel with different dosages of Y_2_O_3_. The results indicated that an appropriate amount of Y_2_O_3_ effectively refined the microstructure and suppressed the precipitation of hard carbide phases. Moreover, the incorporation of rare earth elements enhanced the solid solution’s strengthening effect on the cladding coating, leading to improved wear resistance and electrochemical corrosion resistance of composite coatings. Tao et al. [14] developed a Ni-based alloy coating reinforced by WC micro-particles and nano-Y_2_O_3_ on ultra-high-strength steel. The results confirmed that the addition of nano Y_2_O_3_ inhibited the dissolution of WC during the cladding process. The 2 μm WC-Y_2_O_3_/Ni composite exhibited the lowest friction coefficient. The existing literature indicates that the synergistic effect between WC and Y_2_O_3_ greatly enhances the performance of Ti- and Ni-based laser cladding coatings. However, works have seldom focused on the coating performance of WC/Y_2_O_3_-reinforced Fe-based cladding coatings. Herein, this work investigates the effect of Y_2_O_3_ content on the microstructure, hardness, wear resistance, and corrosion resistance of Fe60/WC cladding coating containing 10 wt% WC. The research result offers significant guidance for fabricating high-performance Fe-based coatings, paving the way for their application in advanced material and component repair.

## 2. Results and Discussion

**Figure 1:**
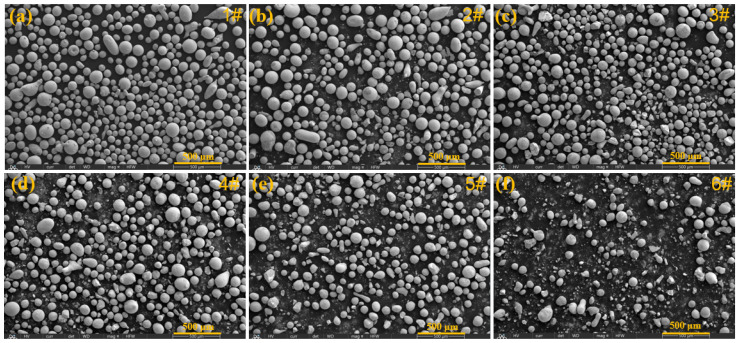
Morphology of various powders: (**a**) Fe60 powders, (**b**) Fe60 + WC, (**c**–**f**) Fe60 + WC + xY_2_O_3_.

Figure 1a shows spherical Fe60 particles with an average particle size of 50–100 μm. Following the method in Table 1, five types of mixed powders were prepared by ball milling for 4h, and their surface morphologies are illustrated in Figure 1b–e. After adding WC particles, the 1# powder in Figure 1b consists of spherical Fe60 particles mixed with irregular WC particles. Figure 1c–e correspond to the 2#–5# powders containing different fractions of Y_2_O_3_. As the Y_2_O_3_ fraction rises, the proportion of spherical particles on the sample surface obviously decreases, while the fine debris increases, indicating the presence of a large number of fine Y_2_O_3_ particles in the mixed powder. Especially for the 5# powder, there are a few big particles and a large number of fine particles, reflecting the effective integration of Fe60, WC, and Y_2_O_3_ particles.

**Figure 2:**
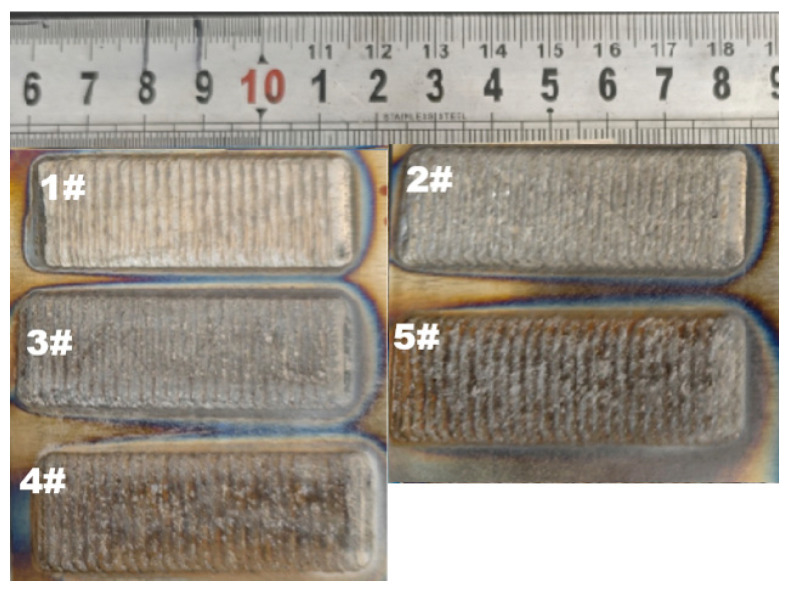
Surface photos of five kinds of laser cladding coatings.

Five types of mixed powders were used for laser cladding onto the surface of 42CrMo steel plate, and the cladding coating is shown in Figure 2. From the 1# sample without Y_2_O_3_, a metallic luster is observed on coating surface. As the amount of Y_2_O_3_ increases, the cladding coating color gradually darkens. When the amount of Y_2_O_3_ is more than 5 wt%, a significant number of pores appear on the surfaces of the 4# coating and 5# coating. The 5# coating shows notably poorer surface quality than the 4# coating. During the laser cladding process, the amount of gas generated in the laser cladding equipment significantly increases with an increase in Y_2_O_3_ dosage due to the decomposition of metal oxides at a high temperature. The decomposed Y_2_O_3_ releases a large amount of oxygen, which reacts with the carbon in Fe60 matrix to form CO_2_ gas. This gas evolution results in the formation of many pores on the 4# and 5# coatings. The presence of extensive pores deteriorates surface quality and decays mechanical properties of the cladding coatings.

**Figure 3:**
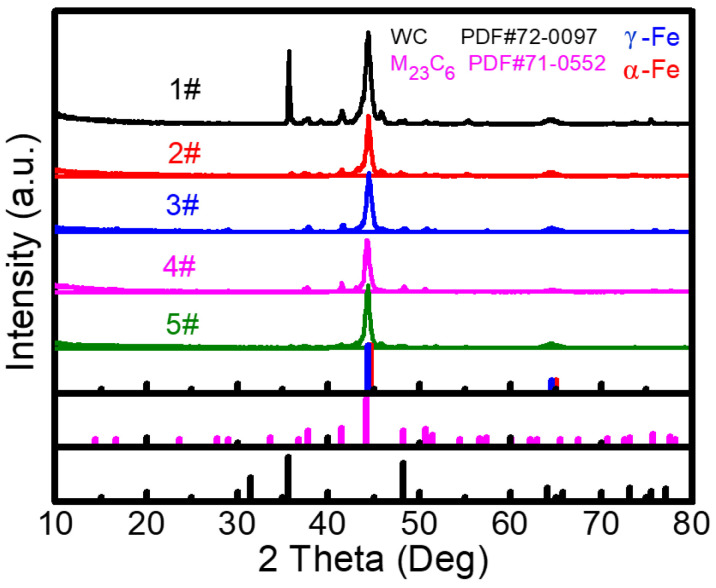
XRD patterns of five kinds of laser cladding coatings.

X-ray diffraction (XRD) analysis was performed to reveal the influence of Y_2_O_3_ dosage on the phase composition and structure of F60/WC cladding coating. In Figure 3, the XRD pattern of the 1# Fe60/WC coating exhibits a strong peak of WC along with weaker peaks of α-Fe and M_23_C_6_ carbides [15], reflecting the presence of residual WC and the generation of new metal carbides. With an increase in Y_2_O_3_ addition, the peak of WC disappears, and the peak intensity of M_23_C_6_ gradually weakens, suggesting that Y_2_O_3_ promotes the decomposition of WC. Furthermore, the addition of Y_2_O_3_ facilitates the phase transition from α-Fe to γ-Fe. When the content of Y_2_O_3_ reaches 7.5 wt%, the peaks of carbides are nearly absent in the 5# sample. The results indicate that a higher Y_2_O_3_ content accelerates the decomposition of WC. During the laser cladding process, the amount of gas generated gradually increases with increasing Y_2_O_3_ content, and the resulting CO_2_ gas release leads to a decrease in carbon content within the molten pool. Consequently, the excessive consumption of carbon reduces the formation of M_23_C_6_ carbides [16].

**Figure 4:**
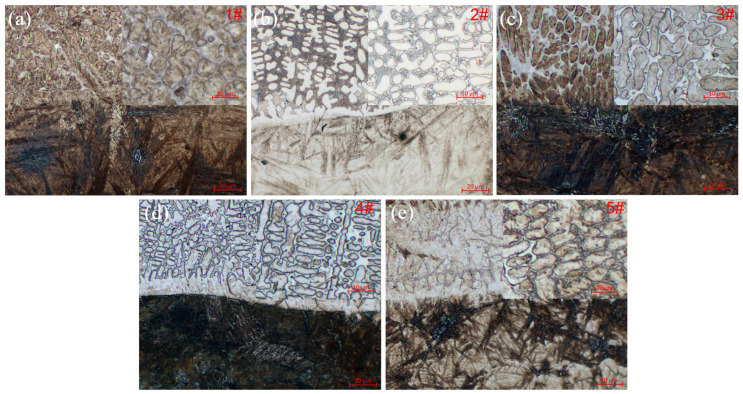
Interfacial region and cladding coating (inset) of the five kinds of laser cladding coatings, (**a**) 1# coating, (**b**) 2# coating, (**c**) 3# coating, (**d**) 4# coating, (**e**) 5# coating.

The metallographic morphologies of the interfacial region and corresponding cladding coating are shown in Figure 4. The lower part of the fusion zone exhibits a Weibull structure, which is a characteristic of the welding overheating zone. The upper fusion layer consists mainly of a eutectic structure. The presence of planar and cellular crystals in the fusion zone of all samples indicates sound metallurgical bonding between the coating and the substrate. The addition of WC in the 1# coating leads to a hypoeutectic structure, which is composed of primary γ-Fe and a eutectic structure. With the introduction of Y_2_O_3_, the eutectic content in the hypoeutectic structure initially increases and then decreases. A more distinct eutectic structure generally corresponds to a higher carbide content. The presence of carbide phases within the γ-Fe contributes to enhanced wear resistance. These results imply that a moderate amount of Y_2_O_3_ effectively promotes eutectic formation, whereas excessive Y_2_O_3_ is detrimental to its development [17]. In Figure 4e, the amount of eutectic structure does not increase further, which is related to the excessive consumption of carbon in the molten pool due to the reaction with Y_2_O_3_, resulting in a reduced carbon content available for carbide formation.

**Figure 5:**
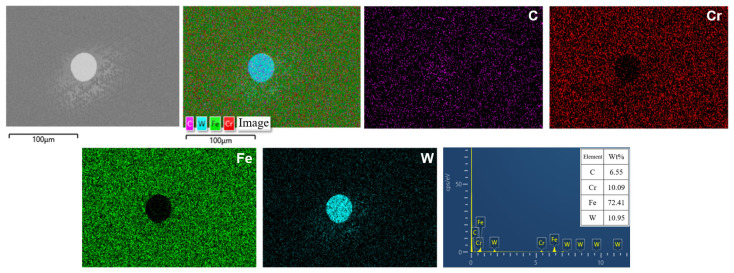
Microstructure and elemental mapping of 1# cladding coating without Y_2_O_3_.

To further analyze the microstructure and element distribution of the laser cladding coating, the morphology of the 1# coating without Y_2_O_3_ is shown in Figure 5. Elemental mapping was conducted in the region containing residual WC particle. It was observed that the white particle was enriched in W and C, indicating the particle identity as residual WC-reinforcing phase. The signals of Fe and Cr in the adjacent matrix region are complementary to these W signals, indicating that WC is embedded within the Fe matrix. The diffusion of W from the bright particle into the surrounding area can be seen clearly, indicating that the WC particle undergoes decomposition during the laser cladding process, and the dissolved W atoms diffuse into the Fe matrix.

**Figure 6:**
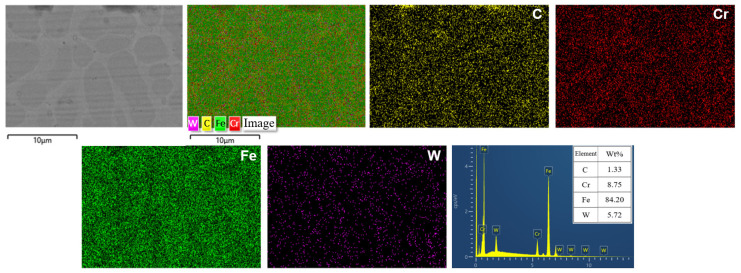
Microstructure and elemental mapping of 3# coating containing 2.5% Y_2_O_3_.

To reveal the effect of Y_2_O_3_ on the microstructure of Fe60/WC coating, Figure 6 provides the morphology and element distribution of the 3# coating with 2.5% Y_2_O_3_. It is difficult to find areas of residual WC on the coating surface, and a grid-like structure can be observed clearly. The distribution map of W reveals that W is mainly concentrated within these grid regions, whereas the depressed areas correspond to the ferrite matrix. This microstructural evolution is attributed to the role of Y_2_O_3_ in promoting the decomposition of WC during laser cladding. The resulting W atoms are uniformly distributed within the newly generated grid framework, thereby reinforcing the Fe matrix. In addition, the C fraction in 3# coating is only 1.33 wt%, much lower than the 6.55 wt% in the 1# coating (Figure 5). This drastic reduction verifies that the addition of Y_2_O_3_ particles greatly decreases the C fraction in the Fe matrix. Furthermore, the overall decrease in the W fraction suggests its successful dissolution and homogeneous dispersion within the Fe matrix.

**Figure 7:**
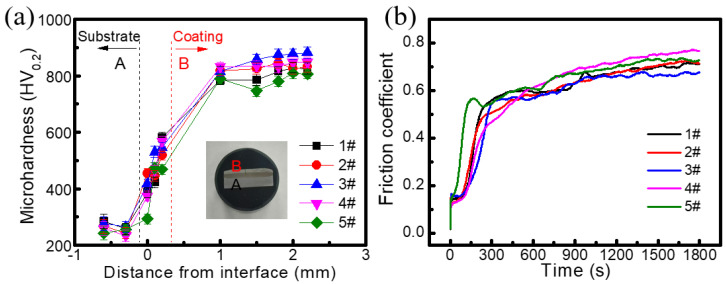
(**a**) Microhardness curves of different coatings upward along the fusion line and (**b**) friction coefficient curves of different coatings.

Figure 7a shows the microhardness curves of different cladding coatings. The hardness of these coatings shows a gradual increasing trend from the steel plate substrate towards the cladding coating, indicating that the cladding coating region possesses superior hardness compared to the substrate. While the substrate hardness remains relatively consistent, the coatings themselves exhibit significant variation. As the amount of Y_2_O_3_ increases, the hardness of the cladding coating shows a general decreasing trend. When the addition amount of Y_2_O_3_ is 2.5 wt%, the 3# coating has the highest average hardness of 861.97 HV. When the addition amount exceeds 2.5 wt%, the hardness of the cladding coating tends to decrease, which is attributed to the increased surface porosity caused by the gas evolution during cladding. In addition to hardness, friction tests were conducted on these cladding coatings by using Si_3_N_4_ balls under a 10 N load, a speed of 120 mm min^−1^, and a stroke of 7 mm. The friction coefficients of different cladding coatings are shown in Figure 7b. During the initial 5 min of the test, all friction coefficient curves fluctuate significantly. During the subsequent wear process, the fluctuation of the curve is alleviated. After 20 min, these fluctuations are mitigated in the subsequent phase and gradually stabilize. The average friction coefficients for coatings 1#–5# are 0.711, 0.713, 0.675, 0.766, and 0.727, respectively. Among them, the 3# coating has a minimum friction coefficient of 0.675, which is in accordance with the highest hardness. Therefore, the 3# coating (Fe60/WC with 2.5 wt% Y_2_O_3_) exhibits the best wear resistance.

**Figure 8:**
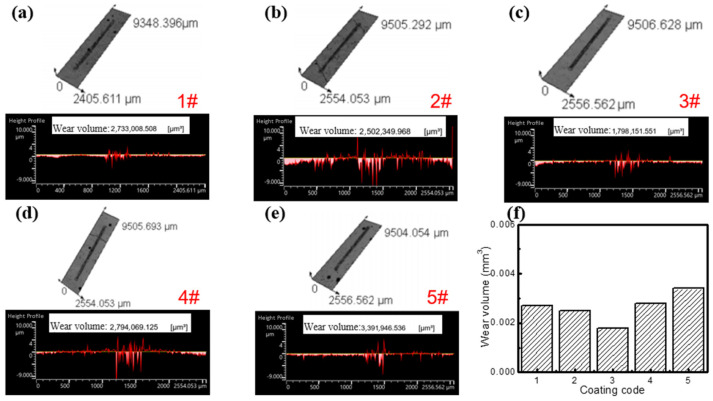
Wear volumes of different coatings and performance comparison, (**a**) 1# coating, (**b**) 2# coating, (**c**) 3# coating, (**d**) 4# coating, (**e**) 5# coating, (**f**) the comparison of different coatings.

The wear volumes of the cladding coating after friction testing were measured by using a laser surface profilometer, as shown in Figure 8. The measured values for coatings 1#–5# are 2.7 × 10^−3^, 2.5 × 10^−3^, 1.8 × 10^−3^, 2.8 × 10^−3^, and 3.4 × 10^−3^ mm^3^, respectively. Among them, the 3# coating presents the smallest wear volume. This result is completely consistent with its minimum friction coefficient, reflecting a positive correlation between wear volume and friction coefficient, further verifying that the Fe60/WC coating with 2.5 wt% Y_2_O_3_ possesses the optimal wear resistance.

**Figure 9:**
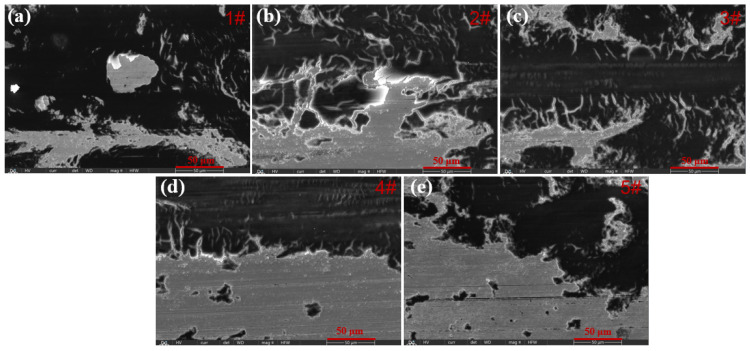
Morphologies of the five kinds of coatings after the friction test, (**a**) 1# coating, (**b**) 2# coating, (**c**) 3# coating, (**d**) 4# coating, (**e**) 5# coating.

Figure 9 shows the worn surface morphologies of the five cladding coatings. The 1# coating exhibits severe abrasive wear, characterized by the presence of large particles and debris particles. This can be attributed to its low hardness, which facilitates material detachment during sliding, thereby accelerating abrasive wear. In contrast, no such particles are observed on the wear region of the other four coatings containing Y_2_O_3_. Among them, the 3# coating shows a relatively smooth surface with only slight plastic deformation, which is consistent with its highest hardness. When the addition amount of Y_2_O_3_ reaches 7.5 wt%, it is difficult to observe wear marks on the worn surface of the 5# coating, which is likely due to the surface pores generated during sample fabricating. These surface pores increase the roughness and promote material loss, which increase its wear amount. The result is consistent with the largest wear volume shown in Figure 8f.

**Figure 10:**
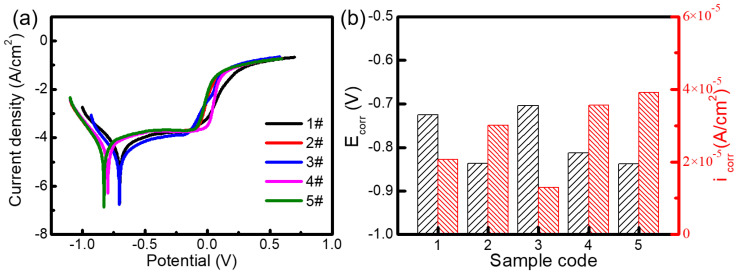
Electrochemical corrosion in 3.5% NaCl solution: (**a**) potentiodynamic polarization curves; (**b**) corrosion potential and corrosion current density.

The polished coating was encapsulated with epoxy resin and served as a working electrode. The dynamic potential polarization (Tafel) curves of different samples were measured in 3.5% NaCl solution [18,19] and are presented in Figure 10a. The corrosion current in the anode region increases with an increase in corrosion potential. There was no obvious anodic passivation zone observed in the five samples, which would have resulted in the direct contact of the electrolyte with the material surface and the formation of pitting corrosion. These Tafel curves were linearly fitted by using Origin software [20,21]. The intersection of two fitted lines was determined by extrapolation and is denoted as the corrosion potential and corrosion current density of the sample. The corrosion potential and corrosion current density of five cladding coatings were obtained and are illustrated in Table 1 and Figure 10b. The corrosion potentials (E_corr_s) of the five coatings are −0.726 V, −0.837 V, −0.704 V, −0.812 V, and −0.838 V, respectively. The corrosion current densities (i_corr_s) of the five coatings are 2.06 × 10^−5^, 3.01 × 10^−5^, 1.30 × 10^−5^, 3.56 × 10^−5^, and 3.91 × 10^−5^ A cm^−2^, respectively. In corrosion analysis, E_corr_ and i_corr_ reflect the corrosion kinetics and thermodynamic properties of materials, respectively. A more negative E_corr_ indicates a higher thermodynamic tendency for corrosion, whereas a larger i_corr_ corresponds to a faster kinetic rate of corrosion. Generally speaking, a higher corrosion potential and a lower corrosion current reflect the superior corrosion resistance of the coating material. Among these coatings, the 3# coating presents the most positive corrosion potential (−0.704 V) and the lowest corrosion current density (1.30 × 10^−5^ A cm^−2^). Therefore, the 3# sample (Fe60/WC with 2.5% Y_2_O_3_) possesses the best corrosion resistance. The optimal corrosion resistance of 3# sample is attributed to the synergistic effect of its improved surface quality and the refined internal microstructure.

**Table 1:** Electrochemical parameters of five coatings obtained from Figure 10a.

Sample	Solution	E_corr_/V_SCE_	i_corr_/A cm^2^
1#	3.5% NaCl	−0.726	2.06 × 10^−5^
2#	3.5% NaCl	−0.837	3.01 × 10^−5^
3#	3.5% NaCl	−0.704	1.30 × 10^−5^
4#	3.5% NaCl	−0.812	3.56 × 10^−5^
5#	3.5% NaCl	−0.838	3.91 × 10^−5^

**Figure 11:**
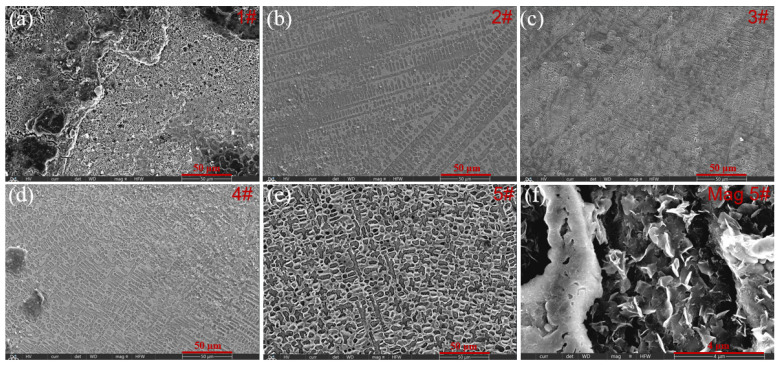
Microstructures of different coatings after the electrochemical test: (**a**) 1# coating, (**b**) 2# coating, (**c**) 3# coating, (**d**) 4# coating, (**e**) 5# coating, and (**f**) magnification of 5# coating.

After electrochemical ×corrosion testing in 3.5% NaCl solution, the surface morphology of different cladding coatings was observed using SEM. In Figure 11a, the surface of the 1# sample (with only WC) displays a limited number of pits alongside moderate corrosion. With an addition of Y_2_O_3_, the surface morphology of the coating undergoes a significant change. Among these samples, the 5# sample shows the most extensive pitting area, mainly due to intergranular corrosion of Fe grains in the pits and producing a large amount of Fe(OH)_3_ corrosion products. Therefore, an excessive amount of Y_2_O_3_ filler ultimately degrades the coating’s corrosion resistance. Through a comparison, the 3# sample containing 2.5% Y_2_O_3_ and 10% WC presents the smallest pitting area, further proving the outstanding corrosion resistance. This observation is in excellent agreement with the electrochemical corrosion data presented in Figure 10b. To further verify the negative impact of a high Y_2_O_3_ fraction on the corrosion resistance of the Fe60/WC coating, Figure 11f provides a high-magnification SEM image of the corroded 5# coating. Abundant layered corrosion products are generated on the sample surface. Both the protruding and recessed areas form a porous structure stacked by Fe(OH)_3_ nanosheets, with a size of several micrometers. The formation o a nanosheet morphology is associated with the excessive consumption of carbon in the molten pool, causing a great decrease in metal carbide faction in the eutectic structure. Consequently, the γ-Fe within the eutectic structure is severely corroded to produce metal hydroxides. In contrast, the 3# sample containing 10% WC and 2.5% Y_2_O_3_ exhibits the smallest pitting area, further demonstrating its superior corrosion resistance in the 3.5% NaCl solution.

## 3. Materials and Methods

### 3.1. Fabrication of Laser Cladding Coatings

Polished 42CrMo steel plate (Xingwang Metal, Dongguan, China) was selected as the substrate material. Commercialized Fe60 powders (Yaoyi Alloys, Shanghai, China) were used as metal matrix, and the components are provided in Table 2. WC powders (~50 μm) and Y_2_O_3_ powders (10~20 μm) were selected as ceramic fillers and modifiers. Mixed powders of Fe60, WC, and Y_2_O_3_ were ball-milled for 4 h by using zirconia spheres. An MFSC 2000W fiber laser device (Chuangxin Laser Co., Ltd., Shanghai, China) was used to prepare laser cladding coating with a power of 1000 W and 2 mm laser spot. The laser cladding process was performed under Ar gas. The scanning speed was 6 mm s^−1^, and the overlap rate was 50%. Synchronous powder feeding was conducted with a gas-powder speed of 10 L·min^−1^. Five kinds of cladding coatings were prepared on 42CrMo steel plate, as illustrated in Table 3.

### 3.2. Material Characterization and Measurement

Scanning electron microscope by Ultra 60 (Zeiss, Shanghai, China) was used for observing the microstructure of the powder and cladding coating, coupled with an energy-dispersive spectrometer (EDS) for measuring the elemental contents. X-ray diffractometer coded as XRD-6100 (Shimazu, Guangzhou, China) was utilized for characterizing the crystal structure and composition, and the scanning rate was 5°·min^−1^. Prior to metallographic structure, the cladding coating needed to be etched by using a dilute HCl/FeCl_3_ solution with a mass ratio of 1/10. Then, anhydrous ethanol was used to rinse the etched surface.

Vickers hardness tester (FM-810, Future-Tech, Tokyo, Japan) was used to measure the coating hardness, in which a 1000 g load was applied on the coating surface for 12 s to obtain the hardness value. Rtec rotary friction testing machine (MFT-5000, Rtec-Instruments, New York, NY, USA) was used to test the friction coefficient of different coatings, and the wear volume was obtained via laser surface profilometer (OLS5100-SAF, Olympus, Tianjin, China). During the friction process, Si_3_N_4_ milling ball was used by applying a 10 N load. The sliding speed was fixed as 120 mm·min^−1^. In corrosion testing, the polished cladding coating was used as a working electrode, and 3.5 wt% NaCl solution was used as an electrolyte [22]. The counter electrode was platinum sheet (1.5 × 1.5 cm^2^), and reference electrode was Calomel electrode. A CHI660E electrochemical workstation (Chenhua 660E, Chenhua, Shanghai, China) was used to measure the potentiodynamic polarization (Tafel) curve of different cladding coatings.

## 4. Conclusions

To fabricate high-performance Fe-based laser cladding coatings, various Fe60/WC/Y_2_O_3_ coatings were deposited on the surface of 42CrMo steel substrates via a laser cladding technique. The influences of the Y_2_O_3_ content on the microstructure, hardness, wear resistance, and corrosion resistance were investigated systematically. The main findings are summarized as follows:

(1) With the addition of Y_2_O_3_, the characteristic diffraction peaks of WC disappear, while the peaks of M_23_C_6_ gradually weaken. This indicates that the introduction of Y_2_O_3_ promotes the decomposition of WC and inhibits the formation of metal carbides. SEM result verifies the formation of abundant grid-like structures in the 3# sample with 2.5 wt% Y_2_O_3_, further demonstrating the uniform distribution of decomposed W within the Fe matrix. When the Y_2_O_3_ content is more than 5 wt%, excessive Y_2_O_3_ introduces a large amount of O, which reacts with C to form CO_2_ gas. This reaction reduces the content of newly generated carbides and increases the number of surface pores.
(2) The dosage of Y_2_O_3_ has a significant impact on the hardness of the cladding coating. The coating hardness initially increases and then decreases with increasing Y_2_O_3_ addition. When the content of Y_2_O_3_ is 2.5 wt%, the 3# sample presents the maximum average hardness of 861.97 HV, which is 3.3 times that of the substrate. The friction coefficient of different cladding coatings was tested using Si_3_N_4_ grinding balls. The 3# sample also exhibits the lowest friction coefficient (0.675) and the smallest wear volume of 1.8 × 10^−3^ mm^3^. Therefore, the Fe60/WC/Y_2_O_3_ cladding coating with 2.5 wt% Y_2_O_3_ demonstrates the optimal wear resistance.
(3) Electrochemical measurement was conducted using polished cladding coating encapsulated with epoxy resin as the working electrode. Tafel curves of different samples were tested. The 3# sample (with 2.5% Y_2_O_3_) presents the most positive corrosion potential (−0.704 V) and the lowest corrosion current density (1.30 × 10^−5^ A cm^−2^). This indicates its superior corrosion resistance in 3.5% NaCl solution, which is attributed to the improved surface quality and the formation of a W-reinforced grid structure.
(4) The incorporation of Y_2_O_3_ additive enhances the interfacial bonding between the metal matrix and WC reinforcement by promoting the decomposition of WC. This microstructural refinement results in a significant improvement in the coating’s hardness, wear resistance, and corrosion resistance. These findings provide a viable strategy for fabricating high-performance composite coatings on steel substrates, which can substantially enhance the durability and reliability of critical equipment components. Consequently, this approach contributes to extending the service life of repaired parts, improving operational reliability, and reducing maintenance costs.

## Figures and Tables

**Table 2:** The components of Fe60 powders.

Fe	C	Cr	Si	Ni	B
60.2 wt%	2.8 wt%	20.5 wt%	3.0 wt%	9.5 wt%	4.0 wt%

**Table 3:** Material contents of different cladding coatings.

Sample Code	Fe60 (g)	WC (g)	Y_2_O_3_ (g)	Y_2_O_3_ Fraction
1#	36	4	0	0%
2#	35	4	0.5	1.3%
3#	34	4	1	2.5%
4#	33	4	2	5.0%
5#	33	4	3	7.5%

## Data Availability

The original contributions presented in this study are included in the article. Further inquiries can be directed to the corresponding author.
